# THE AGING BRAIN: A NEUROVISCERAL INTEGRATION PERSPECTIVE

**DOI:** 10.1093/geroni/igad104.0216

**Published:** 2023-12-21

**Authors:** Julian Thayer

**Affiliations:** University of California, Irvine, Irvine, California, United States

## Abstract

Healthy aging is associated with significant changes in both the brain and the heart. The changes between these, the two most important organs of the body, are linked via the vagus nerve. Using a model of Neurovisceral Integration I will describe how autonomic imbalance and decreased parasympathetic tone in particular may be the final common pathway linking negative affective states and conditions to ill health. The central nervous system network that regulates autonomic balance is closely related and partially overlaps with networks serving executive, social, affective, cognitive, and motivated behavior. This inhibitory cortico-subcortical circuit may structurally as well as functionally, link psychological processes with health-related physiology. When the prefrontal cortex is taken “offline” for whatever reason, parasympathetic inhibitory action is withdrawn and a relative sympathetic dominance associated with disinhibited defensive circuits is released, which can be pathogenic when sustained for long periods. This state is indicated by low heart rate variability (HRV), which is a marker for low parasympathetic activation and prefrontal hypoactivity. In this presentation, I examine the normative changes with aging and the effect that stress may have on how the brain–heart connection changes with age. I will present data linking peripheral physiology to brain structure and function. I conclude by noting that significant sex and ethnic differences exist but that future studies are needed to more fully explicate how they may influence the aging brain and its association with the cardiovascular system.

